# The temporal alignment of mental health consultations across family members: a study of Norwegian adolescents, their parents, and siblings

**DOI:** 10.1007/s00127-024-02803-1

**Published:** 2024-12-11

**Authors:** Jonathan Wörn, Nicoletta Balbo, Karsten Hank, Øystein Kravdal

**Affiliations:** 1 Centre for Fertility and Health, Norwegian Institute of Public Health, Oslo, Norway; 2 Dondena Centre for Research on Social Dynamics and Public Policy, Department of Social and Political Sciences, Bocconi University, Milan, Italy; 3 Institute of Sociology & Social Psychology, University of Cologne, Cologne, Germany

**Keywords:** Intergenerational transmission, Spillover, Teenage, Child, Depression

## Abstract

**Purpose:**

Mental health problems among adolescents have become more prevalent in recent years. Parents’ and siblings’ mental health might be affected by living with a depressed adolescent. This study examines how the mental health of family members develops in the years before and after an adolescent seeks help for depression.

**Methods:**

Unique Norwegian register data that cover the full population are used to estimate models with individual fixed effects. The development in the probability of mental health consultations for parents and older siblings in families with a second-born adolescent seeking help for depression from a GP for the first time is compared to the respective development in families where the second-born adolescent has not had such health care consultation.

**Results:**

Results indicate that adolescents’ depression consultations are associated with a simultaneous increase in mental health consultations in parents and siblings. Mothers and fathers are affected similarly, although the effect seems to be short-lived. Siblings experience a short-term increase in mental health consultations, in addition to a steeper long-term increase across the observation period, compared to peers in families where the second-born adolescent does not seek help for depression. Events that might affect the mental health of multiple family members simultaneously, specifically parental breakup and unemployment, did not explain the observed patterns.

**Conclusion:**

Help-seeking for mental health problems is temporally aligned across family members. Intra- and intergenerational spillovers might contribute to this.

**Supplementary Information:**

The online version contains supplementary material available at 10.1007/s00127-024-02803-1.

## Introduction

Mental health problems affect 10–20% of children and adolescents worldwide [1] and global burden of disease studies identified depression as one of the major causes of health loss [2]. Whereas depression is relatively rare during childhood, adolescence and young adulthood are important life stages for depression onset, with one out of four individuals with depression having experienced first symptoms by the age of 18 [3]. Since the mid-2000s, rates of major depressive episodes among adolescents and young adults have increased substantially [4]. Also in Norway, the prevalence of symptoms of anxiety and depression in adolescents has increased since the mid-1990s, and particularly since the mid-2000s [5].

Mental health problems in adolescence have been shown to predict disadvantages in a variety of adult outcomes for the individuals themselves, such as socio-economic attainment or family formation [6–8]. In addition to implications for the adolescent, there might be implications for their family members. Acknowledging the importance of family ties for individuals’ health [9], this study is concerned with potential intra- and intergenerational spillover effects within the nuclear family. Although previous research has demonstrated intra-familial correlations of mental health [10–13] and shown that individuals’ mental health predicts their family members’ mental health (e.g., 14, 15–17), little is known about the development of mental health in close family members around the time a child in the family experiences a mental health decline so severe that they seek help from medical services. Better knowledge about such co-development of mental health will improve our general understanding of shared health risks in families.

Against this background, this study makes use of longitudinal register data covering the full population of Norway to examine the development of parents’ and siblings’ mental health consultations with a general practitioner (GP) in the years before and after an adolescent child (i.e., a teenager) seeks help for depression from a GP. From teenagers’ perspectives, taking the step of consulting a healthcare professional likely marks a relatively recent decline in mental health that triggers the wish to receive help with a mental state, or reflects another event that triggered help-seeking for a problem that might have existed for a longer time. For the period spanning five years before to five years after teenagers’ first GP-consultation for depression, this study describes changes in their parents’ and siblings’ likelihood of seeking help from a GP for any mental health problem. We use an individual-fixed effects model where we essentially compare the change in a parents’ or siblings’ consultations across time – from before to after the teenager’s consultation – with the corresponding change for parents and siblings of teenagers without such a consultation.

## Background

### Co-development of mental health in children and their parents

It is known that there is a correlation in the risk of having mental health problems between children and their parents. In Norway, adolescents had a 40% higher probability of receiving a (more widely defined) mental health diagnosis if their parent had any (vs. no) sickness absence due to mental health [10]. Other studies report correlations between parent and offspring mental health of about 0.2 [11, 12, 14]. These intergenerational correlations in mental health are typically interpreted as reflecting *‘downward intergenerational transmission’* of health problems from parents to their children, likely through genetic and other biological mechanisms as well as through behaviors and environments [13].

Taking a more longitudinal perspective, some studies focus on whether children’s or parents’ mental health at one timepoint is a stronger predictor of the other parties’ mental health at a subsequent timepoint. Some of these studies report that parental mental health is a stronger predictor of future child mental health than child mental health is of future parental mental health [15, 16]. At the same time, children actively shape their family environment [17], and parents of children with (vs. without) developmental or mental health problems often have more mental health problems [18–21]. This is, in turn, interpreted as support for an *‘upward intergenerational transmission’*, with mental health spillovers from children to parents. Other studies support bi-directional associations between child and parent mental health – although with variation by age and gender [22, 23].

In case of an upward spillover, rising rates of depression in adolescent children might be paralleled by rising rates of mental health problems in parents as well. However, causal directions of potential upward or downward spillovers are hard to establish in the absence of exogenous variation in mental health. This is due to permanent mutual influence between family members over long periods, which makes it hard to identify the family member who experienced the mental health problem that caused the first spillover, unless individuals are observed from birth or even before. Despite of this, there is little doubt that the mental health of children is relevant for the mental health of their parents, and vice versa, if the mental health decline or improvement in a family member is severe enough. In line with this, research exploiting therapy-induced improvement in mental health in one generation in order to assess changes in the mental health of the other generation found that parent’s mental health improves in line with successful treatment for children [24, 25] and that children’s mental health tends to improve when their parents receive successful treatment for their own mental health issues [26, 27]. Nevertheless, little is known about the development in parents’ mental health in the years preceding and succeeding teenagers’ experience of depression.

### Co-development of mental health in siblings

Next to concurrent changes in mental health along intergenerational, vertical lines linking adolescents and their parents, there might also be co-development of mental health along ‘*intragenerational or horizontal’* lines linking teenagers to their siblings. Previous research reported that both internalizing and externalizing behaviors are more frequently found in siblings of children with a (more widely defined) mental health problem [28, 29]. Only very few studies take a longitudinal perspective, with one of them reporting that sibling depressive symptoms predicted adolescent depressive symptoms one year later [30]. Co-development in sibling mental health around the time of teenagers’ depression is thus a largely understudied dynamic that this study addresses by describing the help-seeking for mental health among affected siblings alongside the help-seeking for mental health among affected parents.

### Explanatory mechanisms

We expect that parents and siblings on average experience a decline in mental health (represented by an increase in mental health consultations) around the time their adolescent family member consults a GP for depression. We assume that the adolescent’s mental health decline can trigger mental health declines in parents and siblings through, for example, concerns about their loved one, an increased need for provision of social support, disruption of daily routine, or increases in conflict [31]. It may also be accompanied by other cha(lle)nges in everyday live arising from sharing a household with a child or sibling suffering from milder or more severe depression. Cross-sibling effects might additionally be driven by contagion of unhealthy lifestyles (e.g., lack of physical exercise and sleep, excessive screen time) and reductions in parental attention. In the last couple of decades, spillover effects from children to parents could have been further amplified by the diffusion and deepening of the intensive parenting norm [32], representing the idea that parents’ actions, behaviors and consistent involvement determine children’s healthy development [33, 34]. An alternative mechanism behind cross-family member correlations in mental health might be that the help-seeking behavior of teenagers is part of a process that leads to an improvement in teenagers’ mental health, encouraging other family members to seek help for their own mental health.

### This study

Against this background, this study provides a thorough and systematic description of the co-development of seeking help for mental health problems within families, using full-population, longitudinal register data from Norway. Specifically, the study shows the strength and time-profile of changes in consultations with a GP for mental health problems in parents and siblings in the years before, during, and after teenagers seek help for depression. We believe that this information will further a broader understanding of changes in the health of family systems in the face of adolescents’ depression.

The data contain diagnostic codes from consultations with primary health care services in Norway and pseudonymized information on the identity of close family members. Individual fixed effects models are estimated to control for time-invariant observed and unobserved factors that might affect both the depression risk among the teenagers, their siblings and their parents. Unlike smaller studies that often pooled various mental health and developmental issues, full population data allow us to focus specifically on teenagers seeking help for depression, which is an important driver of the recent decline in young persons’ mental health.

## Method

### Data

Demographic information was extracted from the Population Register of Norway, which includes all persons who have ever lived in Norway since 1964. Everyone is assigned a personal identification number (PIN) that is also used in other registers, and there is information about parents’ PINs, and thus indirectly also about siblings’ PINs. Our second main data source is the Norwegian Control and Payment of Health Reimbursements Database (KUHR), which for the years 2006–2019 includes information about the date of patients’ consultations with GPs and the symptoms and diagnosis code(s) the GPs registered as the reason for consultation. GPs provide this information in order to be reimbursed by the state.

### Sample

We selected families consisting of a mother and father with at least two joint children, where the second-born child (also referred to as “teenager”) was born between 1996 and 1998 (see Online Supplementary Materials Figure A1 for a flow chart of the sample selection). To limit complexity, we further excluded families where any of the parents also had a child with another co-parent. Families where the parents had experienced the death of a child before 2019 were also excluded. If a teenager was registered as being outside the country during any year in the 2009–2016 period (during which we assessed their consultations, see below), the family was not included in the sample. As regards siblings, the focus is on older siblings (i.e., first-born children) because important mental health problems like depression are believed to be underdiagnosed in children, not least because symptom presentation is different in young children [35]. This would limit the possibility to capture potential spillover effects to younger siblings. Parents and siblings were only included in the analyses if they were alive and registered in Norway in at least two years between 2006 and 2019.

### Variables

The main dependent and independent variables refer to consultations with a GP. In the Norwegian public healthcare system, patients are advised to first see their GP if a health problem occurs, and the GP can provide treatment directly when appropriate. The GP also serves an important function in managing sick leave. GPs are equipped to treat depression, using methods like cognitive behavioral therapy and prescribing antidepressants. If a case is too severe or the GP is uncertain about the diagnosis, they will refer the patient to a specialist. Following specialized treatment, the GP receives a summary of the diagnosis and treatment, along with any instructions for ongoing care, if needed. After the specialist treatment is completed, the patient must (again) visit a GP if further help is needed.

Two variables are relevant for *teenage depression*: The first is *whether* the second-born child (“teenager”) had a consultation for depression in the years 2009 to 2016. Teenagers were 11, 12, or 13 years old in 2009 and observed until ages 18, 19, or 20, respectively. A consultation for depression is coded if a consultation registered in KUHR contains the code P76 (International Classification of Primary Care, ICPC-2 system). The second key variable is the *time in years since the teenager had their first depression consultation* (if any), with 0 indicating the year of first consultation. Even though we do not observe individuals from birth, we are confident that we to a large extent capture their first consultation for depression. This is because depression typically has its onset after early teenager years [3]. In own analyses, we found that only 0.8% of individuals that we could observe from their birth in 2006 had a depression consultation before age 13, compared to 9.4% (of the 1999-cohort, observed from age 7) who had such a consultation by the age of 20. For teenagers without a depression consultation (i.e., the control group), we assigned a random year of a mock first depression consultation to allow comparison with the mental health development of family members of teenagers with a depression consultation. The years of mock consultations in ‘control teenagers’ were assigned such that they match the distribution of first consultation years within each birth year cohort of teenagers who did have a consultation for depression. *Any mental health consultation* among parents and siblings is coded if at least one of the mental health diagnosis codes P70-P99 is observed in the year under study (the other codes in the P chapter being symptoms, which we do not consider).

### Method

We estimated linear models for the probability that a family member had any mental health consultation in a given year. Separate models were estimated for the mothers, fathers and siblings of teenagers. The models cover the period spanning from five years before to five years after the teenager’s first depression consultation. The models included the number of years since the teenager’s first (actual or mock) depression consultation (represented by 10 dummy variables), an indicator of whether the teenager had a depression consultation, as well as the interactions between time since consultation and whether there was any consultation. Next to these descriptive models, we estimated models that included individual fixed effects (our main specification).

With such a model, one essentially compares the probability of parental (or sibling) mental health consultations *after* the teenager had a consultation with the corresponding probability for that person *before* the teenager had a consultation, and subtracts the difference that occurs over the same time for a parent (or sibling) whose teenage child (or sibling) has not had a consultation. The difference in development between family members of teenagers with vs. without a consultation for depression can be interpreted as the development that said family member experienced net of ageing and secular changes over time. The underlying assumption is that family members of teenagers with a depression consultation would, in the absence of this consultation, have experienced the same trends in mental health consultations as the family members of teenagers without a depression consultation.

In robustness analyses, we account for union dissolution if the parents were married or cohabiting, for parental unemployment, and for the number and age of the parents’ children. Relevant information was obtained from the Norwegian Population Register and the Tax Database, which were provided by Statistics Norway. We further assess whether our results are robust to various alternative model specifications. Finally, we examine the extent to which the observed patterns in parents’ and siblings’ overall mental health consultations are attributable to parents’ and siblings’ changes in consultations for depression, anxiety, and other mental health problems. To that end, we distinguish between consultations for depression but no other psychological diagnosis (ICPC-2 codes P76 vs. P70-P75/P77-P99), for anxiety but no other psychological diagnosis (P74 vs. P70-73/P75-P99), or any psychological diagnosis but depression or anxiety (P70-73/P75/P77-P99 vs. P76/P74).

## Results

**Fig. 1:**
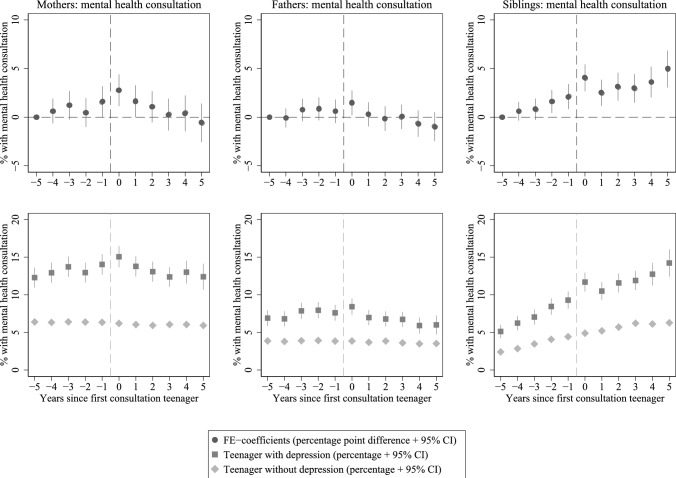
Consultations of mothers, fathers, and first-born siblings with GP for mental health problems, before and after teenagers' (i.e., second-born children’s) first depression consultation. Top row of figures displays the coefficients (percentage point difference) of the linear probability model with individual fixed effects (FE), with year = 0 representing the year the teenager was seeking help for depression. Year = −5 is the reference period. The bottom row of figures displays the estimated share of individuals consulting GPs from linear probability models without control variables, for those with a second-born teenage family member having a consultation for depression in year = 0 (darker squares) and those with a second-born teenage family member that did not have a consultation for depression in the period (lighter diamonds)

The analytical sample consists of approximately 45,000 families and 460,000 family-years, with slight variation across analyses for different family members. Of those families, approximately 2,500 (i.e., approx. 5.5%) experience that their second-born child (“teenager”) consults a GP for depression between 2009 and 2016. Teenagers are observed with a first consultation for depression (if any) at an average age of 17 years, with their mothers being on average 46 years old across the observation period (fathers: 49 years, first-born siblings: 20 years; see Online Supplementary Materials Table A1 – Table A3). In the year following the first consultation for depression, about 33% will have another consultation for depression (see Online Supplementary Materials Figure A2), indicating that some continue seeking help from a GP, while others do not, presumably because the latter experienced that their condition improved, because they stopped seeking help, or because they sought for help elsewhere, and in particular from a specialist. The mothers, fathers, and siblings of teenagers seeking help for depression are approximately twice as likely to have a mental health consultation during the observation period as those in the ‘unaffected’ families (also see Fig. 1).

Our regression analyses reveal two important patterns: Firstly, mothers, fathers, and siblings of teenagers with a consultation for depression exhibit higher probabilities of mental health consultations throughout the observation period spanning from five years before to five years after the first depression consultation for the teenager (Fig. 1, bottom panels; also see Online Supplementary Materials Table A4). Secondly, mothers, fathers, and siblings have a significantly higher probability of having any mental health consultation in the year their teenage family member seeks help for depression, compared to five years before. This is above and beyond any time-constant characteristics of the individual, the family, as well as age- or time-related trends (Fig. 1, top panels; also see Online Supplementary Materials Table A5). The net increase in the probability of mental health consultations relative to five years before the teenager’s consultation was 2.77 percentage points (*p* = .001) for mothers and 1.48 percentage points (*p* = .023) for fathers, corresponding to an increase of 23% and 21%, respectively. The results further suggest that mothers – and to some extent fathers – experience a gradual increase in the probability of any mental health consultation (and thus possibly a decline in mental health) in the years leading up to the teenager’s consultation for depression, and a decline in the years thereafter (possibly reflecting mental health improvements). First-born siblings of second-born teenagers seeking help for depression exhibit a generally steeper increase in the probability of mental health consultations than their counterparts in families without a teenager seeking help for depression, throughout the observation period. This difference in the overall development is likely not a result of the teenager’s depression consultation, but a joint result of the shared environmental and/or genetic predisposition for mental health problems. Two points support this reasoning: (1) Given some degree of transmission across generations [10, 11, 13], the siblings of depression-diagnosed teenagers have a higher risk of mental health problems than their counterparts without a depression-diagnosed sibling; (2) Mental health consultations are relatively rare (or relatively rarely diagnosed [35]) in childhood and become more frequent (or more frequently diagnosed) in adolescence and young adulthood. It is therefore likely that the overall difference in mental health consultations between siblings of teenagers with *versus* without depression – in other words: with a higher or lower family risk for mental health problems – increases as they age. However, the upward deviation from the already steeper age-consultation-trajectory in the year their teenage sibling seeks help for depression indicates that there may be some horizontal spillover – similar to the indications of potential upward spillover to parents. The net increase in siblings’ probability of a mental health consultation in the year of the teenager’s depression consultation relative to five years before the teenager’s consultation is 4.05 percentage points (*p* < .001; 79%), compared to 2.12 percentage points (*p* = .001; 41%) and 2.51 percentage points (*p* < .001; 49%) in the preceding and following year, respectively.

One might expect that parental breakups and parental unemployment could trigger depression diagnoses in all family members at (almost) the same timepoint and thus contribute to the patterns reported above. However, including indicator variables for parental breakup period (i.e., from the year before to the year after the breakup) and parental receipt of unemployment benefits in the relevant year did not affect our results (see Online Supplementary Materials Figure A3). Our analyses were further robust to the addition of (1) age-fixed effects, (2) year*age-fixed effects, (3) year*age-fixed effects plus a time-varying variable for the number of children in age brackets 0–5, 6–11, 11–20, and 20 and more years in the family, and (4) including controls for age without randomly assigning a mock year of depression consultation for the control group (see Online Supplementary Materials Figure A4). Additional analyses showed that for all family members, the increase in their consultations for depression in the year of teenagers’ first depression consultation was about as large as or larger than for all other mental health consultations together (note that years where family members had consultations for more than one of the following causes were not considered in this sub-analysis: depression, anxiety, other psychological diagnoses) (Online Supplementary Materials Figure A5 – Figure A7).

## Conclusion

In 2019, it was estimated that one in seven adolescents worldwide experience mental disorders [36], implying that mental health conditions account for 13% of the global burden of disease among teenagers. If these estimates are the result of an already negative development during the last couple of decades, such trend has further intensified during the COVID-19 pandemic [37, 38]. While there has been a growing recognition of this phenomenon, policy interventions continue to be slow or missing [36].

This study suggests that mental health declines in teenagers might be mirrored in mental health declines in their family members. While previous studies had shown intergenerational correlations of mental health in families [10–12, 14], this study adds that parents and siblings are particularly likely to seek medical help for mental health problems in the year when a teenage family member seeks help for depression. Mothers and fathers show a gradual increase (decline) in the probability of mental health consultations in the years up until (years after) the teenager’s depression consultation. This supports the idea that an (intensified) mental health problem of a teenager also weakens the parents’ mental health. The apparent improvement in parents' mental health some time after the teenager’s depression diagnosis may reflect that the child’s health is improved because of a successful treatment or otherwise, or that the parents somehow adapt to the child’s health problem. An alternative interpretation is that seeking medical help for mental health problems – and possibly experiencing improvement with the situation – encourages other family members to seek such help, for example by raising awareness or increasing confidence in the treatment. This might even be true in the absence of an increase in their experienced disease burden, that is in the case of pre-existing mental health problems [39]. Consequently, visits to GPs might partly represent (a first step towards) improved mental health, and aligned co-development of consultations in families might partly reflect the spread of improvement of mental health in the family. The interpretation in terms of spillovers of mental health rather than spillovers of help-seeking behavior would match well with studies reporting spillover effects in other areas of life. For example, the mental health of partners has been demonstrated to be affected by the other partner’s job loss in quasi-experimental studies [40, 41]. Disentangling the relative importance of the spread of actual mental health problems *versus* the spread of mental health awareness, confidence in treatment, and similar factors, is a task for future studies, which might benefit from combining information on help-seeking behavior with mental health assessments that are independent of help-seeking behavior.

Siblings of teenagers seeking help for depression showed a higher probability of any mental health consultation in the year of the teenagers’ first depression consultation, compared to adjacent years. They further experience a more adverse long-term mental health trajectory compared to their counterparts from families without a second-born child seeking help for depression. While the hump in the year of teenagers' consultation points towards an intragenerational spillover of mental health between siblings, the long-term development probably reflects intergenerational transmission of mental health problems: In the presence of intrafamilial transmission of mental health, an increasing gap in the mental health problems between those from more and those from less mentally healthy families would be expected when larger shares have passed the age of onset for certain diseases (or their diagnosis). While data constraints made us focus on potential spillover effects of second-born children’s depression on first-born children’s mental health, we believe that spillover effects might be stronger from older to younger siblings. This is because older siblings are more likely seen as role models for behaviors, and younger children might be more depending on their parents’ attention and functioning, which might be curtailed by the mental health problems of the sibling.

This study is subject to limitations. Firstly, the absence of exogenous causes of mental health changes in teenagers prevents us from making claims about there being a *causal* effect *of* teenagers' health *on* parents' or siblings' health. This is not only a methodological challenge for most research on this topic, but also a substantive one: Family members do usually spend a lot of time together and depend on each other on a daily basis. In such a context, they are likely to affect each other mutually and constantly. Thus, it is hard to determine whether the reasons that a teenager becomes depressed lie within the teenager themself, or whether (diagnosed or undiagnosed) mental health declines in parents or siblings have triggered the teenager’s mental health decline, which then might feed back into the mental health of their family members. In future studies, the use of higher temporal granularity of mental health assessments could help to shed at least some more light on these issues. Secondly, some family members might visit the same GP or GP-office, and we cannot rule out that information about the patients’ own or their family members’ mental health makes GPs more inclined to discuss mental health problems with family members of the initial patient, resulting in mental health consultations for the family members. This would support arguments of spread of awareness and information as relevant mechanisms, although by means of involvement of other persons than the family members themselves. Thirdly, consultation data from primary care have limited informative value about the duration of mental health problems: Not having any GP-consultation for mental health in the year following the first one might be due to improvement in mental health, referral to specialist care for continued treatment, lack of access to further treatment, or because of giving up on seeking help from the health care system when treatment is not considered effective. Accordingly, the decline in family members’ consultation probability after teenagers’ first depression consultation might be driven by other processes than actual improvements in their mental health, potentially leading to an overestimation of the speed of recovery towards initial levels. This limitation might also lead to an underestimation of the stability of teenage depression. While 33% of teenagers with a consultation for depression had another consultation for depression in the following year, the share of those who still experience problems related to depression might be higher.

To conclude, our analysis has shown temporal alignment of help-seeking behavior within families, both within and across generations. This holds even when confounders like job loss and parental breakups – which might trigger temporary mental health declines in all family members – are controlled for. One plausible interpretation of this pattern is that that mental health declines and improvements in one family member might harm and benefit, respectively, the mental health and wellbeing of other family members. Screening of family members of teenagers with depression might help to identify families in which multiple family members experience or are at risk of having mental health problems. This might be an important step towards improving mental health within these families, and to create more healthy family environments that might further lead to improvements in teenagers’ mental health.

## Supplementary Information

Below is the link to the electronic supplementary material.

Supplementary file1 (PDF 1212 kb)

## Data Availability

The data for this study encompass the Norwegian Population Register and the Norwegian Control and Payment of Health Reimbursements Database, for entire cohorts of the Norwegian population. The authors cannot share these data with other researchers due to their sensitive nature and potential for identification. Researchers can access the data by application to the Regional Committees for Medical and Health Research Ethics and the data owners (Statistics Norway and Norwegian Health Directory, respectively).
