# Supplementary material for: The temporal alignment of mental health consultations across family members: a study of Norwegian adolescents, their parents, and siblings
Source: Soc Psychiatry Psychiatr Epidemiol. 2024 Dec 11;60(7):1671–9. doi: 10.1007/s00127-024-02803-1 (PMC12238148; doi:10.1007/s00127-024-02803-1)
Supplement: Supplementary file 1 — Supplementary file1 (PDF 1212 kb) [file 127_2024_2803_MOESM1_ESM.pdf]

The temporal alignment of mental health consultations across family members: a study of Norwegian adolescents, their parents, and siblings

Jonathan Wörn, Nicoletta Balbo, Karsten Hank, Øystein Kravdal

Corresponding author: Jonathan Wörn, Norwegian Institute of Public Health, jonathan.worn@fhi.no

ONLINE SUPPLEMENTARY MATERIAL: TABLES AND FIGURES

**Table / Figure**	**Page**
Table A 1: Descriptive statistics for the analytical sample of mothers.	2
Table A 2: Descriptive statistics for the analytical sample of fathers.	3
Table A 3: Descriptive statistics for the analytical sample of siblings.	4
Table A 4: Mental health of mothers, fathers, and siblings, by teenager's depression.	5
Table A 5: Fixed effects linear probability models.	6
Figure A 1: Flow chart of sample selection	7
Figure A 2: Probability of teenagers' depression consultation over time.	8
Figure A 3: Models accounting for parental breakup and unemployment.	9
Figure A 4: Robustness check: Different fixed effects-specifications.	10
Figure A 5: Additional analyses: Depression, anxiety, other consultations (mothers).	11
Figure A 6: Additional analyses: Depression, anxiety, other consultations (fathers).	12
Figure A 7: Additional analyses: Depression, anxiety, other consultations (siblings).	13

Table A 1: Descriptive statistics for the analytical sample of mothers.

	**Families with teenager having GP-consultation for depression**					**Control families**				
	Mean/%	*SD*	*Min.*	*Max.*	*Observations*	*Mean/%*	*SD*	*Min.*	*Max.*	*Observations*
Age teenager	16.7	3.3	8	23	25402	16.6	3.3	8	23	434211
Teenager has ever depression consultation	100.0	-	100	100	25402	0.0	-	0	0	434211
Teenager's age at depression consultation	16.9	1.8	11	20	25402	16.9	1.8	11	20	434211
Age mother	46.3	5.4	29	67	25402	46.3	5.3	25	75	434211
Any mental consultation mother	13.3	-	0	100	25402	6.2	-	0	100	434211
Year	2013.5	3.3	2006	2019	25402	2013.6	3.3	2006	2019	434211
Years since teenager's depr. consultation										
-5	9.3	-	0	100	25402	9.3	-	0	100	434211
-4	9.6	-	0	100	25402	9.6	-	0	100	434211
-3	9.8	-	0	100	25402	9.8	-	0	100	434211
-2	9.8	-	0	100	25402	9.8	-	0	100	434211
-1	9.8	-	0	100	25402	9.8	-	0	100	434211
0	9.8	-	0	100	25402	9.8	-	0	100	434211
1	9.7	-	0	100	25402	9.8	-	0	100	434211
2	9.7	-	0	100	25402	9.7	-	0	100	434211
3	9.7	-	0	100	25402	9.7	-	0	100	434211
4	7.4	-	0	100	25402	7.4	-	0	100	434211
5	5.5	-	0	100	25402	5.4	-	0	100	434211
Parental breakup period[a]	6.0	-	0	100	24706	3.7	-	0	100	427241
Any parent unemployed	6.8	-	0	100	21263	6.2	-	0	100	367452

Notes: [a] Breakup period: first year parents are no longer married/cohabiting plus ± 1 year. depr. = depression.

Table A 2: Descriptive statistics for the analytical sample of fathers.

	**Families with teenager having GP-consultation for depression**					**Control families**				
	Mean/%	*SD*	*Min.*	*Max.*	*Observations*	*Mean/%*	*SD*	*Min.*	*Max.*	*Observations*
Age teenager	16.6	3.3	8	23	24959	16.6	3.3	8	23	430146
Teenager has ever depression consultation	100.0	-	100	100	24959	0.0	-	0	0	430146
Teenager's age at depression consultation	16.9	1.8	11	20	24959	16.8	1.8	11	20	430146
Age father	48.8	5.8	31	77	24959	48.9	5.7	28	85	430146
Any mental consultation father	7.2	-	0	100	24959	3.8	-	0	100	430146
Year	2013.5	3.3	2006	2019	24959	2013.5	3.3	2006	2019	430146
Years since teenager's depr. consultation										
-5	9.3	-	0	100	24959	9.3	-	0	100	430146
-4	9.6	-	0	100	24959	9.6	-	0	100	430146
-3	9.8	-	0	100	24959	9.8	-	0	100	430146
-2	9.8	-	0	100	24959	9.8	-	0	100	430146
-1	9.8	-	0	100	24959	9.8	-	0	100	430146
0	9.8	-	0	100	24959	9.8	-	0	100	430146
1	9.7	-	0	100	24959	9.7	-	0	100	430146
2	9.7	-	0	100	24959	9.7	-	0	100	430146
3	9.6	-	0	100	24959	9.7	-	0	100	430146
4	7.4	-	0	100	24959	7.4	-	0	100	430146
5	5.5	-	0	100	24959	5.4	-	0	100	430146
Parental breakup period[a]	6.0	-	0	100	24702	3.7	-	0	100	427216
Any parent unemployed	6.8	-	0	100	21262	6.2	-	0	100	367451

Notes: [a] Breakup period: first year parents are no longer married/cohabiting plus ± 1 year. depr. = depression.

Table A 3: Descriptive statistics for the analytical sample of siblings.

	**Families with teenager having GP-consultation for depression**					**Control families**				
	Mean/%	*SD*	*Min.*	*Max.*	*Observations*	*Mean/%*	*SD*	*Min.*	*Max.*	*Observations*
Age teenager	16.6	3.3	8	23	25515	16.6	3.3	8	23	434265
Teenager has ever depression consultation	100.0	-	100	100	25515	0.0	-	0	0	434265
Teenager's age at depression consultation	16.9	1.8	11	20	25515	16.8	1.8	11	20	434265
Age sibling (firstborn)	20.2	4.0	9	38	25515	19.9	3.9	8	44	434265
Any mental consultation sibling	9.7	-	0	100	25515	4.6	-	0	100	434265
Year	2013.5	3.3	2006	2019	25515	2013.6	3.3	2006	2019	434265
Years since teenager's depr. consultation										
-5	9.3	-	0	100	25515	9.3	-	0	100	434265
-4	9.6	-	0	100	25515	9.6	-	0	100	434265
-3	9.8	-	0	100	25515	9.8	-	0	100	434265
-2	9.8	-	0	100	25515	9.8	-	0	100	434265
-1	9.8	-	0	100	25515	9.8	-	0	100	434265
0	9.8	-	0	100	25515	9.8	-	0	100	434265
1	9.7	-	0	100	25515	9.8	-	0	100	434265
2	9.7	-	0	100	25515	9.7	-	0	100	434265
3	9.7	-	0	100	25515	9.7	-	0	100	434265
4	7.4	-	0	100	25515	7.4	-	0	100	434265
5	5.5	-	0	100	25515	5.4	-	0	100	434265
Parental breakup period[a]	6.0	-	0	100	24567	3.7	-	0	100	424504
Any parent unemployed	6.8	-	0	100	21168	6.2	-	0	100	365700

Notes: [a] Breakup period: first year parents are no longer married/cohabiting plus ± 1 year. depr. = depression.

Table A 4: Share of mental health consultations of mothers, fathers, and siblings of teenagers with (vs. without) consultation for depression. Dependent variable is 100 in case of any consultation and 0 in case of no consultation. Linear probability models.

	**Mothers**		**Fathers**		**Siblings**	
Years since teenager's first (actual or mock) depression consultation (ref: -5 years)						
-4 years	-0.07	[-0.30,0.17]	-0.07	[-0.26,0.11]	0.44***	[0.27,0.61]
-3 years	0.00	[-0.26,0.27]	0.02	[-0.18,0.23]	1.06***	[0.87,1.25]
-2 years	-0.02	[-0.29,0.26]	0.05	[-0.16,0.27]	1.66***	[1.45,1.87]
-1 years	-0.06	[-0.33,0.22]	-0.03	[-0.24,0.19]	2.02***	[1.80,2.24]
0 years	-0.20	[-0.48,0.08]	-0.01	[-0.23,0.21]	2.48***	[2.26,2.71]
1 years	-0.35*	[-0.63,-0.07]	-0.19	[-0.41,0.03]	2.80***	[2.56,3.04]
2 years	-0.47**	[-0.75,-0.18]	-0.02	[-0.25,0.21]	3.30***	[3.05,3.55]
3 years	-0.34*	[-0.63,-0.05]	-0.27*	[-0.49,-0.04]	3.81***	[3.55,4.06]
4 years	-0.34*	[-0.66,-0.02]	-0.38**	[-0.63,-0.13]	3.71***	[3.42,4.00]
5 years	-0.47*	[-0.82,-0.11]	-0.35*	[-0.63,-0.07]	3.87***	[3.54,4.21]
Actual depression consultation (ref: mock)	5.89***	[4.54,7.24]	3.03***	[1.98,4.08]	2.72***	[1.82,3.62]
*Years since teenager's first (actual or mock) depression consultation * actual depression consultation*						
-4 years * dep. consult.	0.71	[-0.57,1.99]	-0.02	[-1.01,0.97]	0.67	[-0.28,1.63]
-3 years * dep. consult.	1.43	[-0.03,2.89]	0.94	[-0.22,2.11]	0.86	[-0.24,1.96]
-2 years * dep. consult.	0.69	[-0.80,2.18]	1.00	[-0.18,2.18]	1.65**	[0.48,2.83]
-1 years * dep. consult.	1.81*	[0.22,3.40]	0.73	[-0.48,1.95]	2.15**	[0.87,3.43]
0 years * dep. consult.	2.96***	[1.31,4.61]	1.53*	[0.24,2.83]	4.08***	[2.70,5.46]
1 years * dep. consult.	1.85*	[0.19,3.50]	0.26	[-1.01,1.53]	2.57***	[1.22,3.93]
2 years * dep. consult.	1.25	[-0.38,2.89]	-0.10	[-1.40,1.21]	3.16***	[1.72,4.60]
3 years * dep. consult.	0.43	[-1.22,2.08]	0.10	[-1.19,1.38]	2.97***	[1.51,4.43]
4 years * dep. consult.	1.06	[-0.82,2.94]	-0.61	[-2.02,0.80]	3.90***	[2.27,5.53]
5 years * dep. consult.	0.58	[-1.45,2.61]	-0.55	[-2.10,1.00]	5.23***	[3.22,7.23]
Constant	6.39***	[6.16,6.63]	3.87***	[3.68,4.06]	2.41***	[2.26,2.56]
Observations	459,613		455,105		459,780	
Individuals	45,020		44,738		45,177	

Notes: 95% confidence intervals in brackets; * $p < 0.05$, ** $p < 0.01$, *** $p < 0.001$.

Table A 5: Linear probability models with individual fixed effects.
Dependent variable is whether the mother, father, or sibling has had any mental health consultation during the year (any consultation=100, no consultation=0).

	**Mothers**	**Fathers**	**Siblings**
*Years since teenager's first (actual or mock) depression consultation (ref: -5 years) * actual depression consultation (ref: mock)*			
-4 years * dep. consult.	0.63	-0.08	0.60
	[-0.65,1.90]	[-1.08,0.91]	[-0.36,1.57]
-3 years * dep. consult.	1.22	0.74	0.81
	[-0.24,2.68]	[-0.42,1.90]	[-0.29,1.91]
-2 years * dep. consult.	0.48	0.85	1.61**
	[-1.01,1.96]	[-0.34,2.03]	[0.43,2.79]
-1 years * dep. consult.	1.59*	0.60	2.12**
	[0.02,3.17]	[-0.60,1.80]	[0.83,3.40]
0 years * dep. consult.	2.77***	1.48*	4.05***
	[1.13,4.41]	[0.20,2.75]	[2.66,5.43]
1 years * dep. consult.	1.63	0.28	2.51***
	[-0.01,3.27]	[-0.96,1.53]	[1.16,3.86]
2 years * dep. consult.	1.06	-0.15	3.14***
	[-0.55,2.68]	[-1.42,1.12]	[1.70,4.58]
3 years * dep. consult.	0.27	0.03	2.96***
	[-1.37,1.90]	[-1.23,1.29]	[1.50,4.42]
4 years * dep. consult.	0.39	-0.68	3.62***
	[-1.46,2.23]	[-2.03,0.68]	[2.04,5.19]
5 years * dep. consult.	-0.59	-0.98	4.95***
	[-2.57,1.39]	[-2.47,0.51]	[3.05,6.86]
Observations	459,613	455,105	459,780
Individuals	45,020	44,738	45,177

Notes: 95% confidence intervals in brackets. * $p < 0.05$, ** $p < 0.01$, *** $p < 0.001$. dep. consult. = depression consultation.

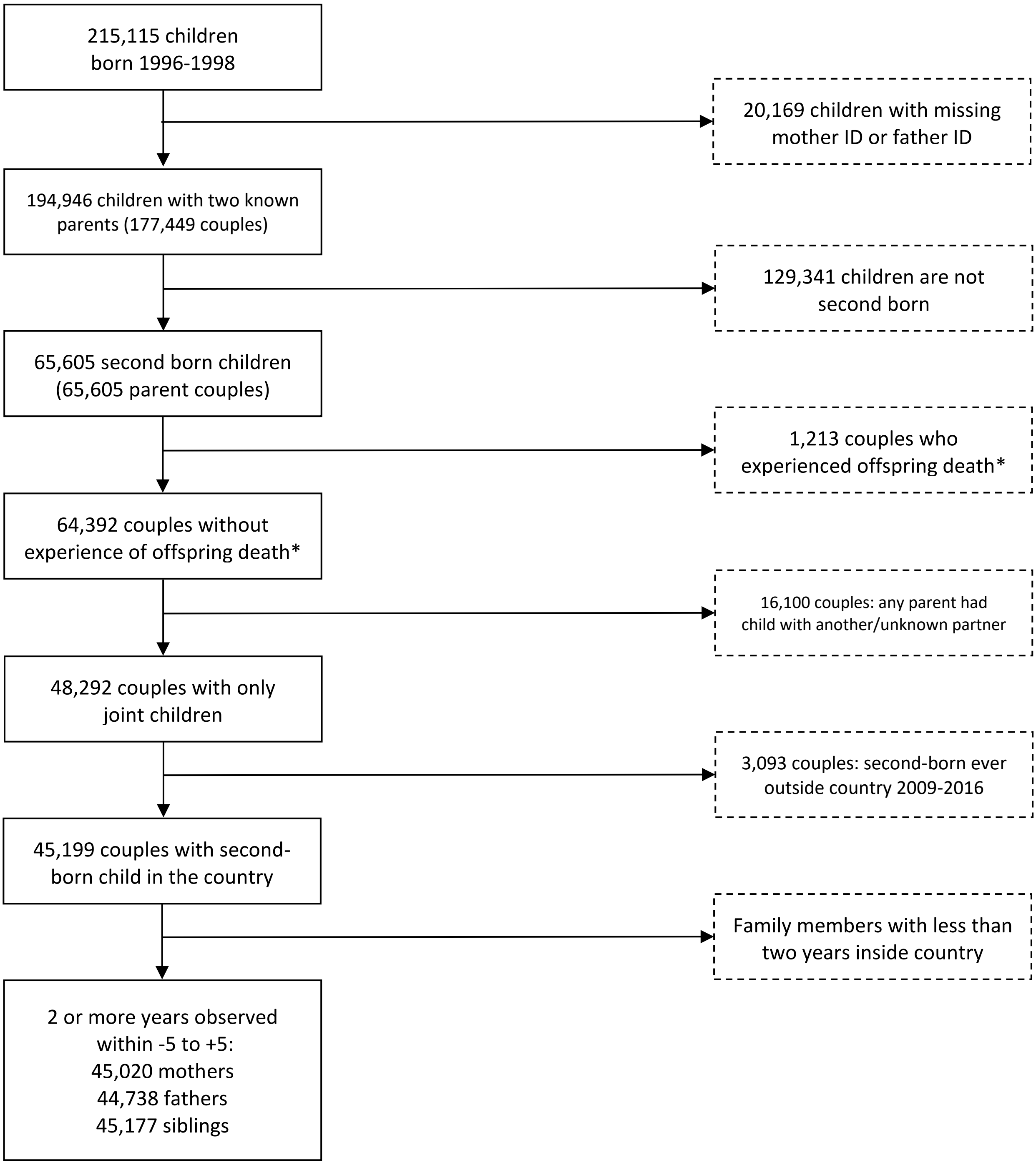

Figure A 1: Flow chart of sample selection
Notes: * before 2019

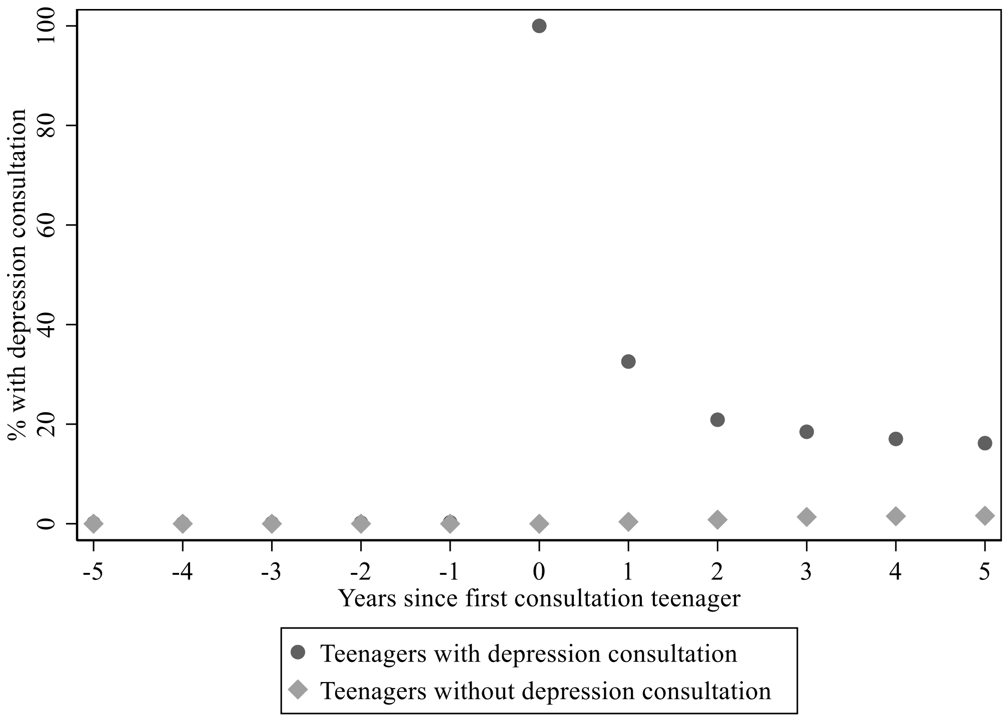

Figure A 2: Probability of teenagers' depression consultation over time. By years since the first (actual or mock) depression consultation, for teenagers with a depression consultation and control teenagers without a depression consultation.

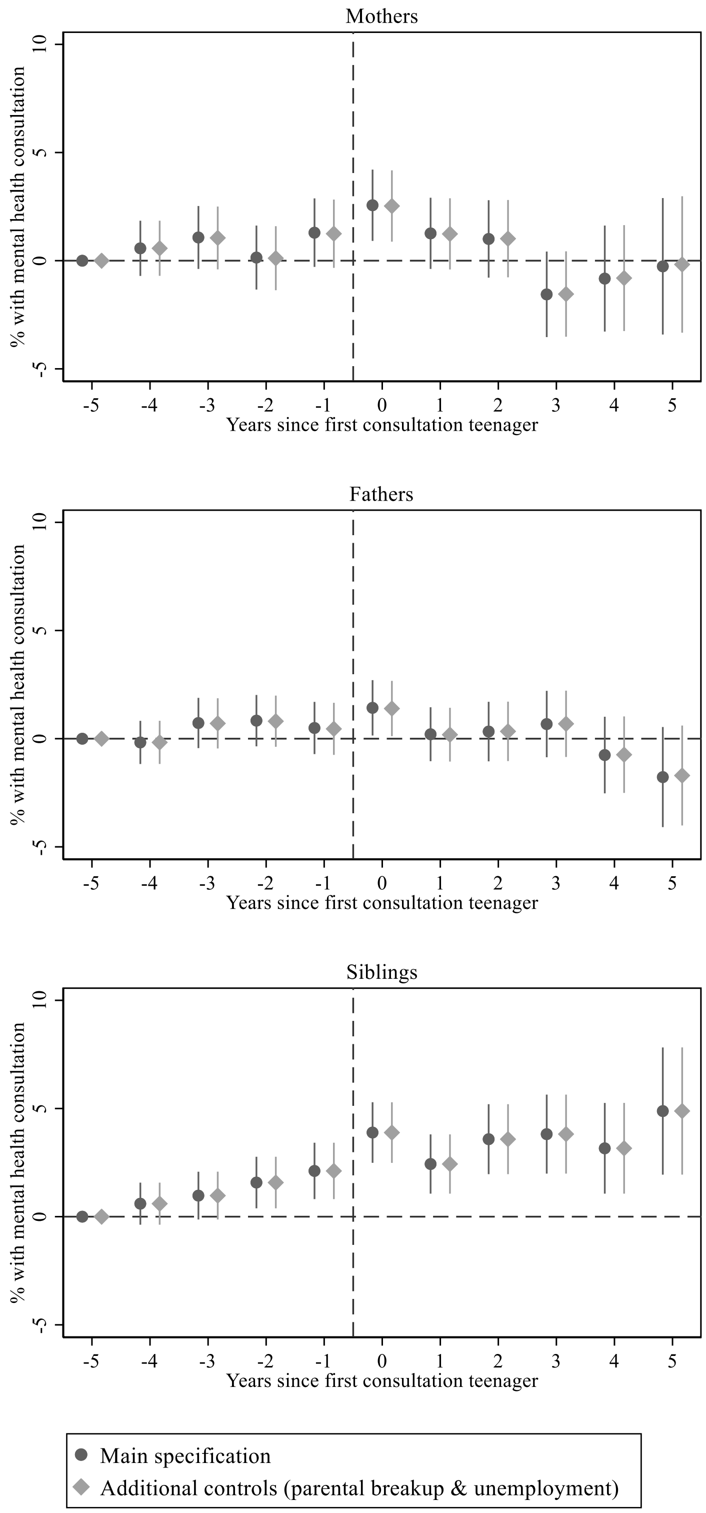

Figure A 3: Models with and without control variables for parental breakup and unemployment. Coefficients from linear regression models individual fixed effects, with year -5 before the teenager's first depression consultation as the reference category. Graphs include the main specification (as reported in the manuscript) and a model with additional controls (an indicator for the period spanning the year before to the year after parental breakup and an indicator for receipt of unemployment benefit of any parent in the year). Note that the sample for these analyses differs slightly from the original analyses. This is foremost due to availability of employment data until 2017 in our dataset and because only parents that were cohabiting or married were at risk of breakup.

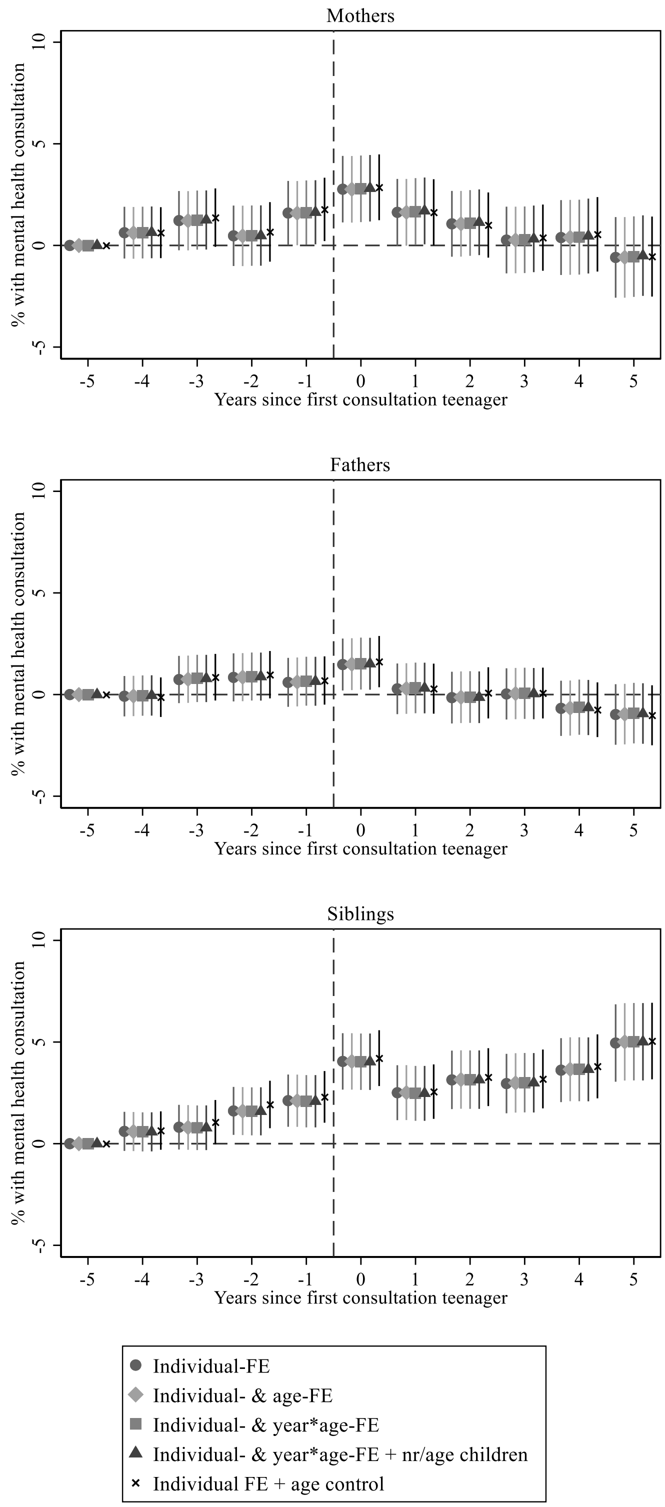

Figure A 4: Different specifications of linear probability models with individual fixed effects. Coefficients from individual fixed effects linear regression models with year -5 before the teenager's first depression consultation as the reference category. Models 1-4: Individual fixed effects (main specification); individual and age fixed effects; individual and year*age fixed effects; individual and year*age fixed effects plus the number of children in age brackets 0-5, 6-11, 12-19, and 20 and older. Model 5: Fixed effects for individual, and controls for age, with control individuals not assigned a random year of teenagers' first mock consultation. Instead, control individuals are assigned a constant value on "years since consultation" and contribute to the estimation of the coefficient for age.

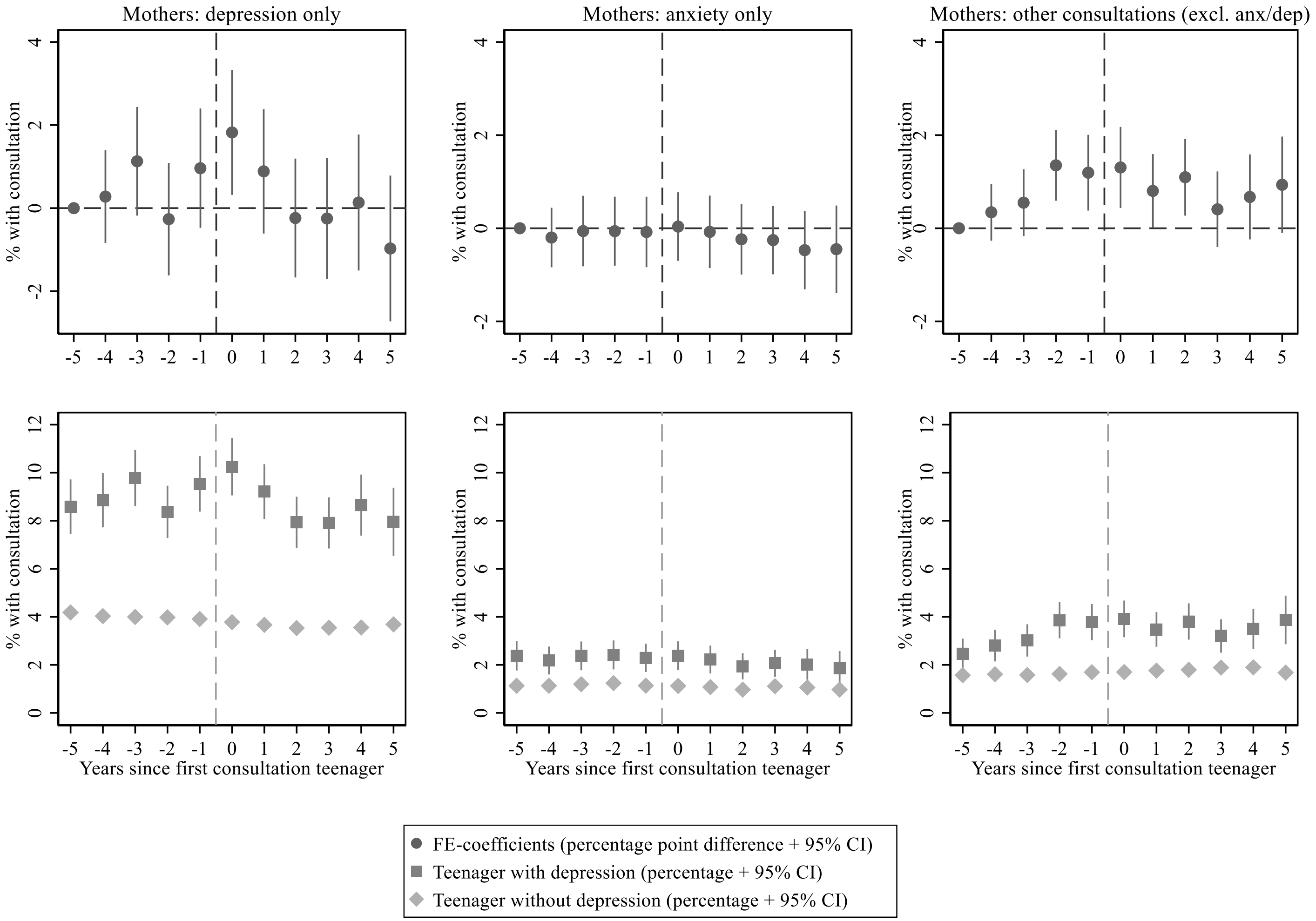

Figure A 5: Depression, anxiety, and other psychological consultations (mothers). Consultation of mothers with GP for depression, anxiety, and all other psychological diagnoses, before and after the teenager's first depression consultation. Top row of figures displays the coefficients (percentage point difference) from the linear probability model with individual fixed effects, with year = 0 representing the year the teenager had their first consultation for depression. year = -5 is the reference period. The bottom row of figures displays the share of individuals consulting GPs as estimated from linear probability models without control variables, for those with a teenage family member *with* a depression consultation (darker squares) and those with a teenage family member *without* a depression consultation (lighter diamonds).

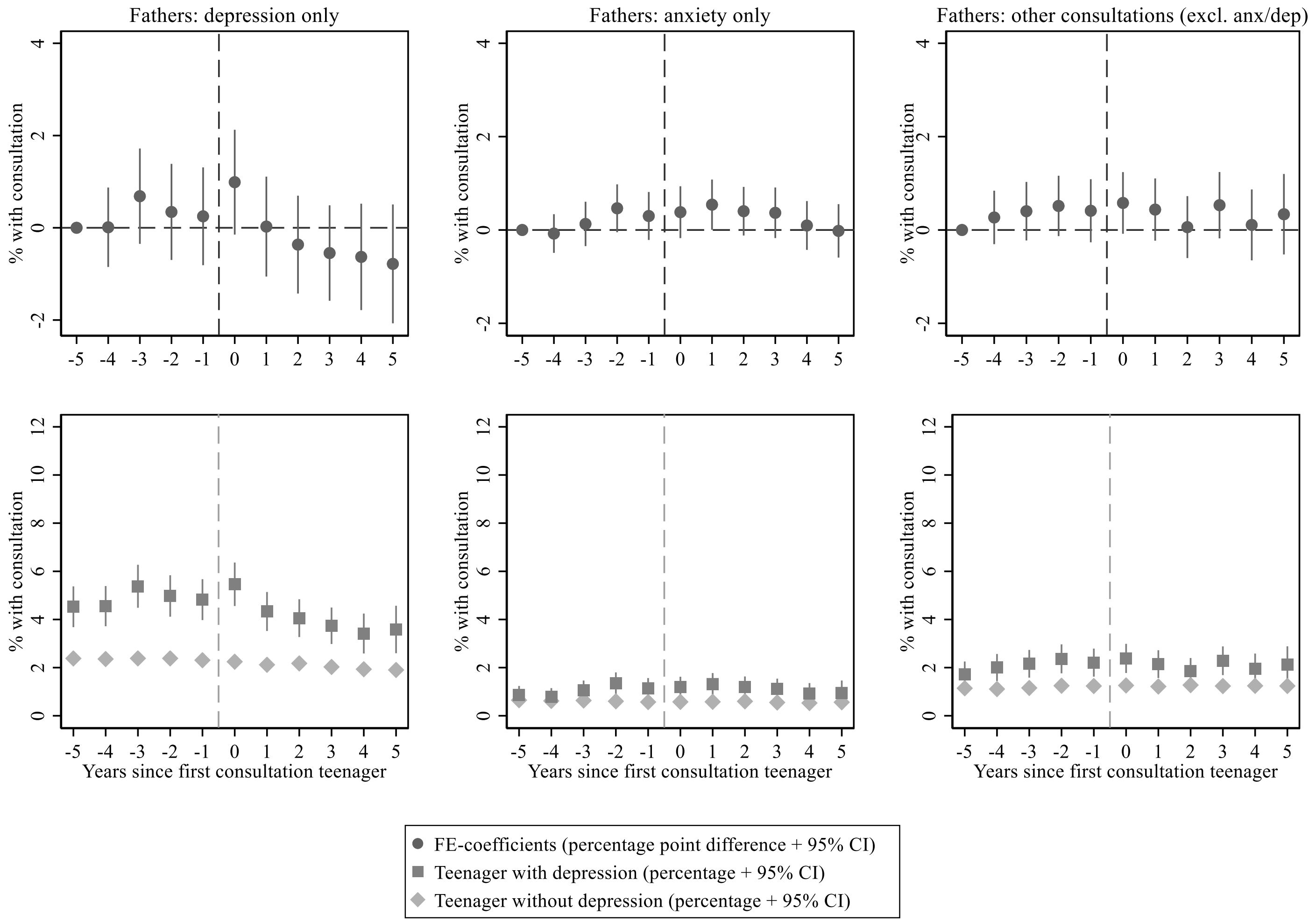

Figure A 6: Depression, anxiety, and other psychological consultations (fathers). Consultation of fathers GP for depression, anxiety, and all other psychological diagnoses, before and after the teenager's first depression consultation. Top row of figures displays the coefficients (percentage point difference) from the linear probability model with individual fixed effects, with year = 0 representing the year the teenager had their first consultation for depression. year = -5 is the reference period. The bottom row of figures displays the share of individuals consulting GPs as estimated from linear probability models without control variables, for those with a teenage family member *with* a depression consultation (darker squares) and those with a teenage family member *without* a depression consultation (lighter diamonds).

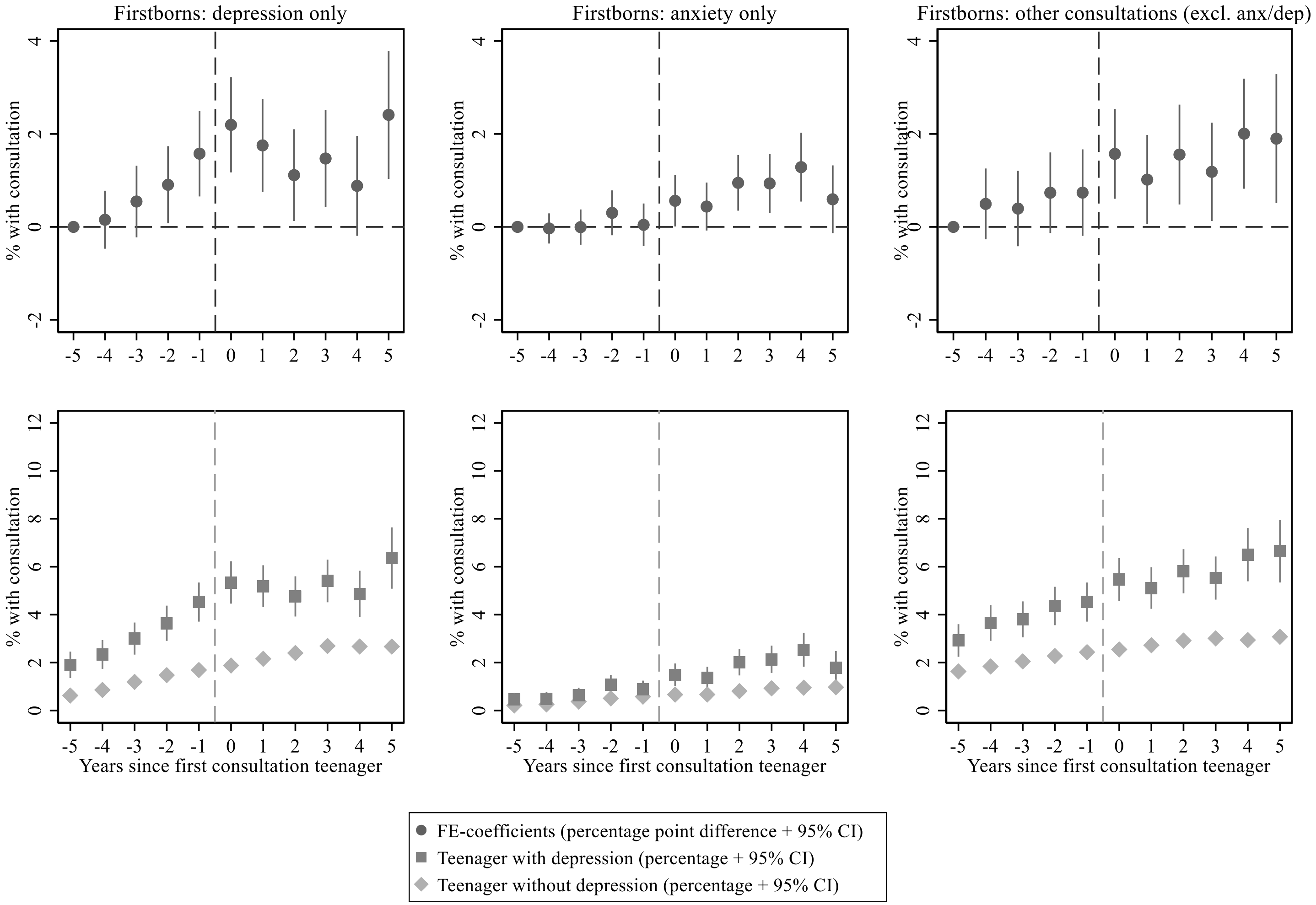

Figure A 7: Depression, anxiety, and other psychological consultations (siblings). Consultation of firstborn siblings with GP for depression, anxiety, and all other psychological diagnoses, before and after the teenager's first depression consultation. Top row of figures displays the coefficients (percentage point difference) from the linear probability model with individual fixed effects, with year = 0 representing the year the teenager had their first consultation for depression. year = -5 is the reference period. The bottom row of figures displays the share of individuals consulting GPs as estimated from linear probability models without control variables, for those with a teenage family member *with* a depression consultation (darker squares) and those with a teenage family member *without* a depression consultation (lighter diamonds).
